# Molecular Identification of *Sarcocystis* Species in Sheep from Lithuania

**DOI:** 10.3390/ani12162048

**Published:** 2022-08-11

**Authors:** Alina Marandykina-Prakienė, Dalius Butkauskas, Naglis Gudiškis, Evelina Juozaitytė-Ngugu, Vytautas Januškevičius, Eglė Rudaitytė-Lukošienė, Petras Prakas

**Affiliations:** Nature Research Centre, Akademijos Str. 2, LT-08412 Vilnius, Lithuania

**Keywords:** *Sarcocystis arieticanis*, *Sarcocystis tenella*, domestic sheep, *cox1*, molecular identification, epidemiology

## Abstract

**Simple Summary:**

Members of the genus *Sarcocystis* (Apicomplexa: Sarcocystidae) are protozoans that have a two-host, prey–predator life cycle. These parasites are most thoroughly examined in economically important animals. Several *Sarcocystis* species are known to form macroscopic and microscopic sarcocysts in the muscle tissues of sheep. A previous study carried out in Lithuania has shown a maximum *Sarcocystis* infection prevalence in sheep and a high parasite burden; however, species were not identified. In the present study, diaphragm, oesophagus, and heart samples of 69 sheep raised in Lithuania were examined. No macrocysts were detected in the sheep muscles, while the microcysts found corresponded to two morphological types. By molecular methods, two *Sarcocystis* species, *S*. *arieticanis* and *S*. *tenella*, using canids as their definitive hosts, were identified for the first time in sheep from Lithuania. Both species were detected in all the studied animals. In summary, very high detection rates of *S*. *arieticanis* and *S*. *tenella* ranging from 82.61% to 100% were established in the examined diaphragm, oesophagus, and heart muscle samples of sheep.

**Abstract:**

Data on the distribution of different *Sarcocystis* species in various muscles of sheep are scarce. In the present study, 190 diaphragm, oesophagus, and heart muscle samples of 69 sheep raised in Lithuania were examined for the presence of *Sarcocystis* spp. Under a light microscope, two morphological types of microcysts corresponding to *S*. *arieticanis* and *S*. *tenella* were detected. Eight and 12 sarcocysts of *S*. *arieticanis* and *S*. *tenella*, respectively, were isolated and characterised by the sequencing of a portion of *cox1*. The sequence comparisons revealed the highest similarity between European and Asian isolates of *S*. *arieticanis* and *S*. *tenella* obtained from domestic sheep and other wild Caprinae hosts. Based on peptic digestion, nested PCR targeting cox1, and sequencing, a 100% infection prevalence of *S*. *arieticanis* and *S*. *tenella* was observed in the 69 studied animals. The occurrence of *S*. *tenella* was significantly higher in the diaphragm than in the oesophagus (χ2 = 13.14, *p* < 0.001), whereas differences in the prevalence of *S*. *arieticanis* in the studied muscle types were insignificant (χ2 = 1.28, *p* > 0.05). Further molecularly based epidemiological studies are needed to compare the prevalence of *Sarcocystis* species in various muscles of sheep raised in different geographic regions.

## 1. Introduction

Representatives of the genus *Sarcocystis* (Apicomplexa: Sarcocystidae) are protozoans widespread globally in mammals, birds, and reptiles. These parasites are characterized by a two-host, prey–predator life cycle. Sarcocysts are formed mainly in the muscles of the intermediate host, while sporocysts develop in the small intestine of the definitive host. The intermediate host gets infected through contaminated food or water, and the definitive host is infected by ingesting mainly muscular tissues containing mature sarcocysts [1].

Economically important animals, such as cattle, sheep, goats, and pigs, have most comprehensively been examined for *Sarcocystis* infection [2]. Four *Sarcocystis* species, *S*. *arieticanis*, *S*. *tenella*, *S*. *gigantea*, and *S*. *medusiformis*, using sheep (*Ovis aries*) as intermediate hosts, have been characterized most comprehensively [3]. The first two species (*S*. *arieticanis* and *S*. *tenella*) form microcysts in muscle tissues and are transmitted by canids, whereas *S*. *gigantea* and *S*. *medusiformis* produce macrocysts and are transmitted by felids [4]. Other species described in sheep are rare; *S*. *microps* [5] and *S*. *mihoensis* [6] were reported in China and Japan, respectively. Based on transmission experiments, dogs were found to act as definitive hosts for these two poorly investigated species [5,6]. However, a later phylogenetic study demonstrated that *S*. *mihoensis*-like presumably use corvids and other scavenger birds as definitive hosts [7]. Furthermore, *S. gracilis*-like sarcocysts were found in single sheep from Italy [8].

In most cases, *Sarcocystis* spp. are non-pathogenic for sheep [9]. However, acute *S*. *arieticanis* and *S*. *tenella* infections can lead to fever, loss of appetite, anaemia, and abortion or the premature birth of lambs [10]. Furthermore, contamination of sheep carcasses with macroscopic sarcocysts results in significant losses in the animal husbandry industry [11]. Therefore, it is important to conduct and develop epidemiological studies on *Sarcocystis* spp. In sheep.

Thus far, five *Sarcocystis* species from sheep, *S*. *arieticanis*, *S*. *gigantea*, *S*. *medusiformis*, *S*. *mihoensis*, and *S*. *tenella*, have been investigated by means of genetic methods. It was shown that the mitochondrial cytochrome c oxidase subunit I gene (*cox1*) was the best choice for the discrimination of these *Sarcocystis* species [7]. *Sarcocystis* species from sheep are usually identified by sequencing [3,4,7,9,10,12,13,14,15,16]. By contrast, in some studies *Sarcocystis* species from sheep have been confirmed by PCR-RFLP [17,18].

It was shown that as compared to morphological methods, molecular approaches were superior in detecting *Sarcocystis* species [19]. Typically, *Sarcocystis* species were identified by isolating sarcocysts from muscles and characterizing them genetically [7]. However, isolation of sarcocysts is ineffective in large-scale epidemiological studies [20]. By contrast, muscle digestion combined with PCR assays may be a rapid and accurate technique for detecting *Sarcocystis* species [21].

In Lithuania, such farm animals as sheep, cattle, pigs, and horses were examined by morphological methods for *Sarcocystis* occurrence. The highest *Sarcocystis* infection prevalence and intensity were determined in sheep [2]. In Lithuania, *Sarcocystis* species were previously identified only in cattle, revealing the presence of four species, including zoonotic *S*. *hominis* [20]. The aim of the present study was to identity *Sarcocystis* species in sheep from Lithuania by means of combined digestion and molecular methods and to compare the distribution of *Sarcocystis* species detected in diaphragm, oesophagus, and heart samples.

## 2. Materials and Methods

### 2.1. Sample Collection

Tissue samples were collected from 69 sheep immediately after the slaughter process. In total, 190 muscle tissue samples from the diaphragms, oesophaguses, and hearts of sheep were collected. Whole organs were collected for the detection of *Sarcocystis* spp. Diaphragm and oesophagus samples were collected from 17 sheep, while diaphragm, oesophagus and heart samples were taken from 52 animals. The examined animals were raised in north-eastern Lithuania (54–55° N, 24–26° W) and were collected from a slaughterhouse in the village of Alanta, Molėtai district, during the period lasting from October 2019 to October 2020. The ages of the inspected animals were determined by registration numbers in the Government system. Forty sheep were under a year old, while six sheep were from one to two years old, eight sheep were three years old, and 15 sheep were older than three years. 

### 2.2. Microscopic Examination of Sarcocysts

All tissue samples were transported to the Nature Research Centre, the Laboratory of Molecular Ecology (Vilnius, Lithuania), and were kept frozen (–20 °C) until a microscopical and digestion examination was conducted. A visual inspection of the collected sheep tissues for macroscopic sarcocysts of *Sarcocystis* spp. was repeatedly carried out at the laboratory. Afterwards, sarcocysts were examined in freshly squashed muscle samples under the Nikon ECLIPSE 80 i light microscope (Nikon Corp., Tokyo, Japan). Overall, 70 sarcocysts were isolated from the muscle fibres of the diaphragm and oesophagus with the help of two preparation needles. These sarcocysts were morphologically characterised according to the size and shape of the cysts, the structure of the sarcocyst wall, and the size of bradyzoites located inside the cyst. The morphology of bradyzoites was inspected by gently pressing the cover slip, rupturing the sarcocyst wall, and releasing bradyzoites. Eleven sarcocysts isolated from the diaphragm and nine sarcocysts isolated from the oesophagus were preserved in individual 1.5 mL microcentrifuge tubes containing 50 μL 96% ethanol and kept frozen at −20 °C for a further molecular characterisation.

### 2.3. Molecular and Phylogenetic Analysis of Sarcocysts

For the identification of *Sarcocystis* species and evaluation of intraspecific genetic variability, isolated sarcocysts of two morphological types, twelve sarcocysts having finger-like protrusions, and eight sarcocysts characterised by hair-like protrusions were subjected to molecular examination. DNA extraction of twenty sarcocysts (C2.2, C5.2, C14.1, C14.2, C28.1, C28.2, C34.2, C40.2, C55.1, C57.1, and C57.2 extracted from diaphragm samples, and C2.1, C5.1, C10.1, C10.2, C18.1, C18.2, C34.1, C40.1, and C55.2 extracted from oesophagus samples) isolated from ten sheep individuals was performed using a GeneJET Genomic DNA Purification Kit (Thermo Fisher Scientific Baltics, Vilnius, Lithuania), according to the manufacturer’s recommendations. The final DNA concentration in the solutions was measured with a spectrophotometer after the DNA extraction. DNA concentrations in samples reached up to 15 ng/μL.

Obtained DNA samples were later used for the PCR amplification of *cox1* sequences. Partial *cox1* sequences were amplified using the SF1/SsunR3 primer pair listed in Table 1. Amplification was carried out in a 25 μL reaction mixture containing 12.5 μL of DreamTaq PCR Master Mix (Thermo Fisher Scientific Baltics, Vilnius, Lithuania), 5 μL of DNA template, 0.5 μM of both forward and reverse primers, and nuclease-free water up to 25 μL. The PCR cycling conditions started with 5 min at 95 °C, followed by 40 cycles of 45 s at 94 °C, 60 s at 60 °C, and 80 s at 72 °C, and ended with 10 min at 72 °C. The evaluation of PCR products was made using 1% agarose gel electrophoresis, and 5 μL of each PCR product was purified with alkaline phosphatase FastAP and exonuclease ExoI (Thermo Fisher Scientific Baltics, Vilnius, Lithuania) to remove unincorporated nucleotides and primers. 

Purified PCR samples were sequenced using a Big-Dye^®^Terminator v3.1 Cycle Sequencing Kit (Thermo Fisher Scientific, Vilnius, Lithuania) and a 3500 Genetic Analyzer (Applied Biosystems, Foster City, CA, USA), according to the manufacturer’s instructions. Sequencing was performed in both directions using the same forward and reverse primers as for PCR. Sequencing results were validated by comparing the obtained sequences with those of various *Sarcocystis* spp. by Nucleotide BLAST search (http://blast.ncbi.nlm.nih.gov/, accessed on 6 June 2022).

Phylogenetic analysis was conducted to evaluate the intraspecific genetic relatedness of identified Sarcocystis species by comparison of isolates of the same species obtained from different intermediate hosts and various countries. Sequences for the analysis were chosen based on previous phylogenetic studies [7,22] and the results of the Nucleotide BLAST comparison. Sequences exported from the GenBank database were aligned with those obtained in the current study. Multiple sequence alignments were generated with the MUSCLE algorithm available in MEGA7 software [23]. MEGA7 was used to select a nucleotide substitution model and to construct phylogenetic trees using the maximum likelihood method. The bootstrap method with 1000 bootstrap replications was used to test the robustness of the phylogeny.

### 2.4. Peptic Digestion

All 190 muscle samples were used for *Sarcocystis* species identification by combined peptic digestion and the nested PCR (nPCR) technique. During the preparation of each sample, several pieces of muscle were cut off from different parts of the organ. Then, 25 ± 3 g of each muscle sample was minced and subjected to peptic digestion according to a modification of the protocol for isolating *Toxoplasma gondii* from animal tissues for the isolation of *Sarcocystis* [26]. Minced samples were blended with 150 mL of saline. Afterwards, 150 mL of acid pepsin solution containing 1.04 g pepsin, 2 g NaCl, and 7 mL HCl (pH 1.10–1.20) was prewarmed (37 °C) and added to the homogenate. The suspensions were placed in an orbital shaking incubator for 2 h at 37 °C and were later filtered into individual flasks using a strainer. Collected filtrates were then transferred to sterile centrifuge tubes and centrifuged at 1200× *g* for 8 min. The supernatant was poured off and the procedure was repeated until there was no residual liquid left. After removing the supernatant, an additional 50 mL of saline was added, and the solution was centrifuged at 1200× *g* for 8 min. Fluid from the sediment was poured off and the samples were stored at −4 °C until genomic DNA extraction.

### 2.5. Molecular Analysis of Digested Samples

Genomic DNA from the digested samples was extracted using a PureLink Microbiome DNA Purification Kit (Invitrogen by Thermo Fisher Scientific, Waltham, MA, USA), according to the manufacturer’s instructions. After peptic digestion and DNA extraction, the concentration of the resulting DNA was measured. The final DNA concentration ranged from 19.1 to 83.4 ng/μL. Some samples were diluted with elution buffer so that the final DNA concentration did not exceed 40 ng/μL. In order to identify *Sarcocystis* species, all isolates were used for species-specific PCR amplification with the PCR primers listed in Table 1. Primers were designed with the help of the Primer 3 plus program [27].

The first amplification step of nPCR was performed under the same conditions as are used in conventional PCR. The second amplification step was carried out in 25 μL reaction mixture containing 12.5 μL of DreamTaq PCR Master Mix (Thermo Fisher Scientific Baltics, Vilnius, Lithuania), 2 μL of DNA template, 0.5 μM of both forward and reverse internal primers, and nuclease-free water up to 25 μL. Cycling conditions for the first amplification step were the same as those described above, except for the 56–60 °C annealing temperatures used, depending on the primer pair. The second nPCR amplification cycling conditions started with 5 min at 95 °C, followed by 35 cycles of 45 s at 94 °C, 45 s at 59–62 °C, and 60 s at 72 °C, and ended with 7 min at 72 °C. The annealing temperatures used depended on the primer pair. A total of three negative controls were used: one for the first amplification step and two for the second amplification step. The second negative control for the second amplification step was obtained by transferring 2 μL from the negative control of the first amplification step to the negative control of the second amplification step. Positive DNA controls were obtained in the present study by isolating sarcocysts of *S. arieticanis* and *S. tenella*. However, positive DNA controls for *S. gigantea*, *S. medusiformis*, and *S. mihoensis* were not obtained. Amplified PCR products were visualized by 1% agarose gel electrophoresis.

The PCR purification and sequencing procedures used were the same as those described above. Despite the fact that, in some cases, not only products of the second nPCR step but also longer products of the first amplification step were visible in agarose gels, the chromatograms were pure without double peaks. The sequences obtained were compared using the BLAST program. For the BLAST analysis, ≥90% query coverage was set. In the present study, generated *cox1* sequences of *Sarcocystis* species from sheep were deposited in GenBank with the accession numbers ON858956–ON859017.

## 3. Results

### 3.1. Morphology of Sarcocysts as Observed under a Light Microscope

During the initial examination of the muscle samples with the naked eye, no macrocysts were observed either while collecting them at the slaughterhouse or while re-testing them at the laboratory. By contrast, two types of microcysts corresponding to *S*. *arieticanis* and *S*. *tenella* were observed under the light microscope.

**Figure 1:**
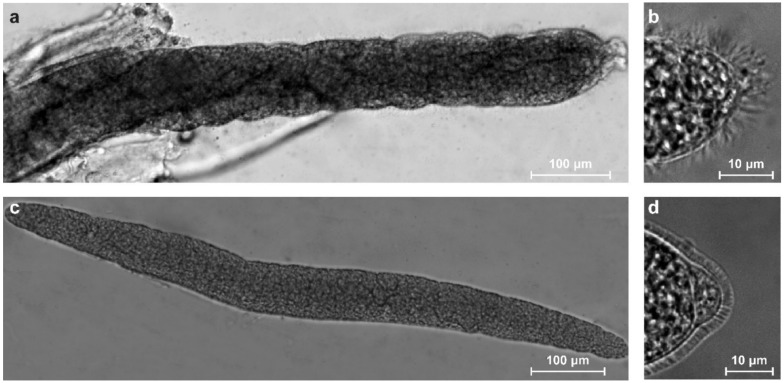
Morphologies of *S*. *arieticanis* and *S*. *tenella* isolated from the diaphragm of sheep as observed under a light microscope. Fresh preparations: (**a**,**b**) *S*. *arieticanis*; (**c**,**d**) *S*. *tenella*. (**a**) Fragment of ribbon-shaped sarcocyst incorporated in muscle tissues. (**b**) A portion of sarcocyst wall with irregularly arranged hair-like protrusions. (**c**) A spindle-shaped sarcocyst released from muscle tissues. (**d**) A portion of sarcocyst wall with densely packed finger-like protrusions.

In the diaphragm and oesophagus samples, sarcocysts of *S*. *arieticanis* were ribbon-shaped, measuring 470–1693 × 75–130 μm (999 ± 316 × 92 ± 13 μm; *n* = 30) in size (Figure 1a). The sarcocyst walls had numerous thin irregularly arranged hair-like protrusions, which were 4.2–7.3 μm (5.8 ± 0.8 μm; *n* = 30) in length (Figure 1b). During manipulation of the sarcocysts, the fragile hair-like protrusions were torn in some cases. Cysts were septate, and their interior compartments were filled with banana-shaped bradyzoites measuring 9.5–14.0 × 2.5–4.8 μm (11.7 ± 1.1 × 3.3 ± 0.4 μm; *n* =150) in size.

In the diaphragm and oesophagus samples, sarcocysts of *S*. *tenella* were spindle-shaped, measuring 350–1350 × 28–130 μm (705 ± 235 × 70 ± 24 μm; *n* = 40) (Figure 1c). The sarcocyst walls were 2.0–4.5 μm (3.1 ± 0.6 μm; *n* = 40) in thickness and had numerous very densely packed finger-like protrusions (Figure 1d). The interiors of the sarcocysts were filled with banana-shaped bradyzoites that measured 9.5–14.7 × 2.9–5.2 μm (12.7 ± 1.3 × 3.8 ± 0.5 μm; *n* = 250).

### 3.2. Molecular and Phylogenetic Analysis of Isolated Sarcocysts

A standard PCR using the SF1/SSunR3 primer pair and subsequent sequencing was successful for 20 sarcocysts analysed. Twelve of the isolated sarcocysts were assigned to *S*. *tenella* and the remaining eight were attributed to *S*. *arieticanis*. The 894 bp-long *cox1* sequences of *S*. *arieticanis* showed 99.11–100% similarity to each other, differing by up to eight single-nucleotide polymorphisms (SNPs), whereas a comparison of the 894 bp-long *cox1* sequences of *S*. *tenella* demonstrated 98.66–100% similarity to each other and up to 12 SNPs. Eight *S*. *arieticanis* sequences comprised 5 haplotypes, while 12 *S*. *tenella* sequences were divided into 11 haplotypes.

**Figure 2:**
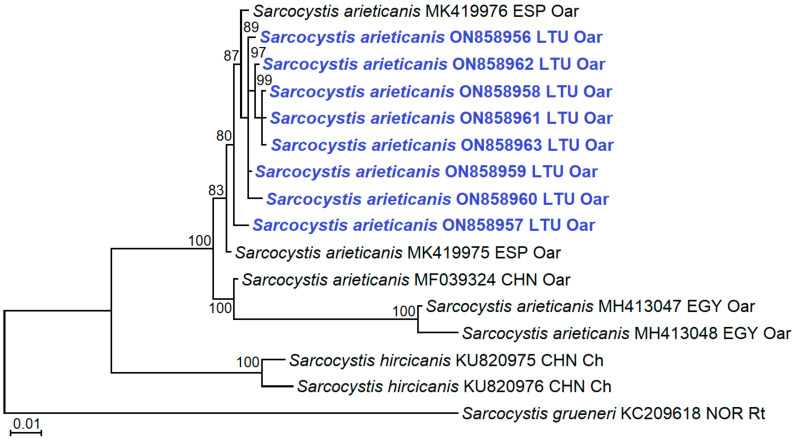
Phylogenetic trees of selected *Sarcocystis* spp. based on *cox1* sequences showing genetic relatedness of isolated sarcocysts to *S*. *arieticanis*. The tree was rooted on *S*. *grueneri*. The figures next to the branches show the bootstrap support values. Sequences obtained in the present study are marked in blue. The final alignment consisted of 3 taxa, 16 sequences, and 894 aligned nucleotide positions. The Kimura 2-parameter + G model was chosen for the phylogenetic analysis. CHN: China, EGY: Egypt, ESP: Spain, LTU: Lithuania, NOR: Norway, Ch: Capra hircus, Oar: Ovis aries, Rt: Rangifer tarandus.

In the present study, the obtained sequences of *S*. *arieticanis* displayed a high similarity (98.77–99.78%) to three sequences of *S*. *arieticanis* from domestic sheep in Spain and China (Table 2). By contrast, only 92.39–93.85% sequence similarity was observed when comparing sequences obtained in this work with two Egyptian isolates (MH413047–MH413048). In the phylogram, eight isolates of *S*. *arieticanis* obtained in the current study were placed together with *S*. *arieticanis* isolates from domestic sheep in Spain and formed a sister branch to Chinese and Egyptian isolates of *S*. *arieticanis* (Figure 2). The phylogenetic analysis revealed that *S*. *arieticanis* was a sister species to *S*. *hircicanis* from domestic goats.

**Figure 3:**
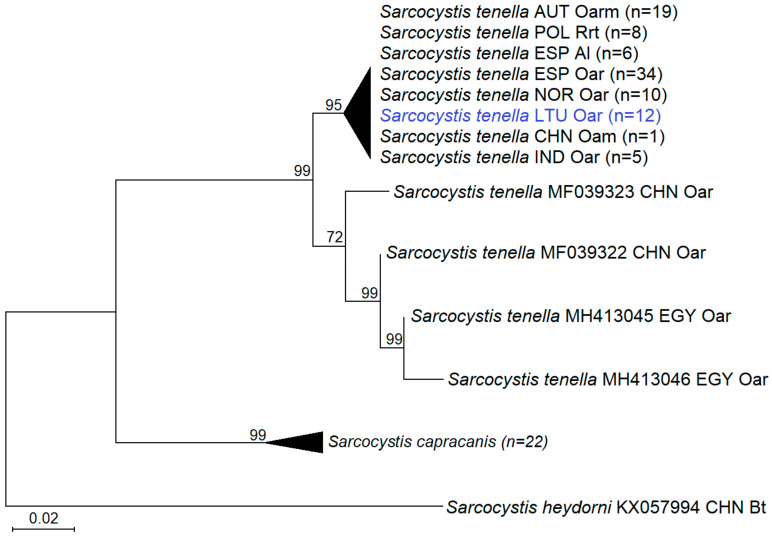
Phylogenetic trees of selected *Sarcocystis* spp. based on *cox1* sequences showing the genetic relatedness of isolated sarcocysts to *S*. *tenella*. The tree was rooted on *S*. *heydorni*. The figures next to the branches show the bootstrap support values. Sequences obtained in the present study are marked in blue. The final alignment consisted of 3 taxa, 122 sequences, and 863 aligned nucleotide positions. The Kimura 2-parameter + G + I model was chosen for the phylogenetic analysis. AUT: Austria, CHN: China, EGY: Egypt, ESP: Spain, IND: India, LTU: Lithuania, NOR: Norway, POL: Poland, Al: *Ammotragus lervia*, Bt: *Bos taurus*, Oar: *Ovis aries*, Oarm: *Ovis aries musimon*, Oam: *Ovis ammon*, Rrt: *Rupicapra rupicapra tatrica*, *n*: number of sequences.

The analysis of intraspecific genetic variability of *S*. *tenella* showed that genetic differences within *cox1* did not depend on the intermediate host of the parasite (Table 2 and Figure 3). High sequence similarity values (97.32–99.89%) were obtained by comparing *S*. *tenella* isolates from Lithuanian domestic sheep with those from domestic sheep, European mouflon (*Ovis aries musimon*), Tatra chamois (*Rupicapra rupicapra tatrica*), Barbary sheep (*Ammotragus lervia*), and argali (*Ovis ammon*) from Austria, Poland, Spain, Norway, China, and India (Table 2). Meanwhile 95.86–97.54% sequence similarity was determined by comparing *S*. *tenella* from Lithuanian domestic sheep with *S*. *tenella* from domestic sheep in Egypt. In the phylogenetic tree, 95 out of 99 sequences of *S*. *tenella* were grouped into a cluster with high support (95 bootstrap value). This cluster consisted of *S*. *tenella* isolates obtained from various intermediate hosts, argali, barbary sheep, domestic sheep, mouflon, and Tatra chamois. In the present study, the analysed *S*. *tenella* isolates from Lithuania were also placed in this clade. Four remaining *S*. *tenella* sequences were acquired from Chinese and Egyptian domestic sheep. Based on partial *cox1* sequences, *S*. *tenella* was separated from the most closely related *S*. *capracanis*, which used the domestic goat as intermediate host.

### 3.3. Identification of Sarcocystis spp. in Sheep from Lithuania by nPCR

Using primers designed for the identification of *S. tenella* and *S. arieticanis*, fragments corresponding to the theoretical sizes were successfully amplified. The use of DNA controls derived from sarcocysts proved that the primer pairs V2arie3/V2arie4 and V3tenF3/V3tenR2 were specific to *S. arieticanis* and *S. tenella*, respectively. By contrast, no amplification was obtained with primers theoretically targeting *S*. *gigantea*, *S*. *medusiformis*, and *S*. *mihoensis*.

Further sequencing was employed to verify that *S. tenella* and *S. arieticanis* species could be identified from the resulting fragments. In this way, 21 amplified fragments for each species, with 7 fragments from the diaphragm, oesophagus, and heart, were randomly selected and subject to sequencing. The obtained sequences were trimmed, excluding the primer binding sites. Thus, 325 bp-long *S*. *arieticanis* (corresponding to 430–754 sites in *S*. *tenella* MK420009) and 338 bp-long *S*. *tenella* (corresponding to 532–869 sites in *S*. *tenella* MK420009) sequences were analysed. It should be concluded that *S. arieticanis* and *S. tenella* can be reliably identified by the technique described in the present study, as the obtained intraspecific and interspecific genetic similarity values did not overlap (Table 3). The sequence comparison showed that the barcoding gap was wider for *S*. *tenella* than for *S*. *arieticanis*.

### 3.4. Distribution of Sarcocystis spp. in the Examined Sheep Samples from Lithuania

By digestion and nPCR, 100% infection prevalence for *S*. *arieticanis* and *S*. *tenella* was observed in the 69 animals studied. The maximum *Sarcocystis* spp. prevalence was detected in the diaphragm and heart, while a 97.10% infection rate was determined in the oesophagus (Table 4). In general, the prevalence of *S*. *arieticanis* and *S*. *tenella* in the analysed muscles ranged from 82.61% to 100%. Comparing the detection rate of two *Sarcocystis* species, *S*. *tenella* was more frequently found in the diaphragm and heart, and *S*. *arieticanis* was more common in the oesophagus; however, the differences observed were statistically insignificant (*p* > 0.05). The occurrence of *S*. *tenella* differed significantly between the three muscle types analysed (χ^2^ = 14.80, df = 2, *p* < 0.01). The differences were due to a significantly higher (χ^2^ = 13.14, df = 1, *p* < 0.001) prevalence of *S*. *tenella* in the diaphragm (100%) than in the oesophagus (82.61%). By contrast, the detection rate of *S*. *arieticanis* in the examined muscle types did not differ significantly (χ^2^ = 1.28, df = 2, *p* = 0.575). Mixed infections with *S*. *arieticanis* and *S*. *tenella* were significantly more frequently (χ^2^ = 8.42, df = 1, *p* < 0.01) observed in the diaphragm (94.20%) than in the oesophagus (76.81%), whereas in the heart, mixed infections occurred in 82.69% of cases. Observed differences in *Sarcocystis* species prevalence were not significant (χ^2^ ≤ 1.95, df = 2, *p* ≥ 0.163) in two analysed age groups of sheep (younger than two years and older than two years). In conclusion, a very high infection prevalence of both identified species, *S*. *arieticanis* and *S*. *tenella*, was established in the diaphragm, oesophagus, and heart muscles of sheep raised in Lithuania.

## 4. Discussion

### 4.1. Prevalence of Sarcocystis spp. in Sheep

Previously, in Lithuania, based on methylene-blue staining of squashed muscle samples of 61 studied domestic sheep, 100% *Sarcocystis* spp. prevalence was detected in oesophagus, diaphragm, heart, jaw, neck, and back muscle samples [2]. On the basis of a molecular analysis, the present study revealed 100% *Sarcocystis* spp. prevalence in the diaphragm (69/69) and heart (52/52) and a 97.10% infection rate (67/69) in the oesophagus. Across the globe, *Sarcocystis* spp. prevalence rates in sheep vary from 9.0 to 100%, depending on the method used [1]. In the current work, *S. arieticanis* and *S. tenella* producing microscopic sarcocysts were identified in all 69 examined animals. In general, the detection rates of microscopic *S. arieticanis* and *S. tenella* is higher as compared to the observation of macrocysts of *Sarcocystis* spp. worldwide [9,10,30]. For instance, some recent studies described occurrence rates of microscopic *Sarcocystis* spp. greater than 70% in sheep from Brazil [12], China [4], Egypt [14], Iran [16,31,32], Iraq [33,34], and Malaysia [35], whereas the highest prevalence of macroscopic *Sarcocystis* spp. in sheep was reported in Iran, reaching up to 37% [16,17,32,36,37]. *Sarcocystis gigantea* is assumed to be a more frequent macroscopic species as compared to *S. medusiformis* [17,18,38].

### 4.2. Canids Serve as Definitive Hosts of Sarcocystis spp. in Sheep from Lithuania

In the present study, the *Sarcocystis* species *S. tenella* and *S. arieticanis*, transmitted by canids, were detected. These species are known to be more pathogenic as compared to other *Sarcocystis* species found in sheep which are transmitted via felids [7,10,13,14,15,16]. Non-detection of macrocysts in sheep was an expected outcome of the current study, since local slaughterhouse veterinarians note that macrocysts in sheep are rare, occurring every few years (personal communication with veterinarian Rimas Rudėnas). High prevalence rates of *S. tenella* and *S. arieticanis* can be explained by the fact that sheep farmers keep dogs to protect their herds. As a result, farmed sheep can freely interact with the faeces of canids [1]. The defecation behaviour of dogs may only facilitate the spread of *Sarcocystis* spp. sporocysts, which leads to infection. On the other hand, the limiting factor of exposure to feline faeces is that cats tend to bury their excrement [38,39]. Furthermore, macroscopic cysts of *S. gigantea* and *S. medusiformis* tend to require longer time periods (from 1 to 2 years) to grow and fully develop [39,40,41]. Although we investigated 23 adult (over 2 years) individuals, no macroscopic cysts were found.

### 4.3. Genetic Characterisation of Sarcocystis spp. in Sheep

Nuclear *18S* rRNA, *28S* rRNA, *ITS1*, and mitochondrial *cox1* have been examined in *Sarcocystis* spp. from sheep, and the latter gene has been demonstrated to be most appropriate for species identification [4,7]. Thus far, numerous *cox1* sequences of *S*. *tenella* have been available from Austria, China, Egypt, India, Norway, Poland, and Spain [3,4,7,9,14,24,25,28,29]. By contrast, only six *cox1* sequences of *S*. *arieticanis* from domestic sheep in China, Egypt, and Spain [4,7,14] and from the European mouflon in Austria [25] could be found in GenBank. Here, we obtained twelve and eight 894 bp-long *cox1* sequences of *S*. *tenella* and *S*. *arieticanis* from sheep in Lithuania, respectively. Additionally, relatively high intraspecific variation within *cox1* of these two *Sarcocystis* species was demonstrated (Table 2, Figure 2 and Figure 3). Hence, the accumulation of *cox1* sequences of *S*. *arieticanis* and *S*. *tenella*, representing isolates from different geographical regions and hosts, is important for the development of species identification techniques which could be applied globally.

### 4.4. Distribution of Sarcocystis Species in Different Muscles of Sheep

In the current work, *Sarcocystis* species were identified by peptic digestion combined with nPCR. *Sarcocystis arieticanis* detection rates in the diaphragm, oesophagus, and heart were 94.20%, 91.30%, and 88.46%, respectively, whereas *S*. *tenella* was identified in all diaphragm samples examined, in 94.23% of the heart samples, and in 82.61% of the oesophagus samples. Significant differences were noticed in comparing the detection rates of *S*. *tenella* in the diaphragm and oesophagus (χ^2^ = 13.14, df = 1, *p* < 0.001). It should be noted that data on the prevalence of the following *Sarcocystis* species in various muscles are scarce. According to Metwally et al. (2019) [15], *S. tenella* was more frequent in the diaphragm (62/91, 68.13%) and heart (45/91, 49.45%) as compared to the oesophagus (24/91, 26.37%) and tongue (22/91, 24.18%) in domestic sheep raised in Saudi Arabia. The prevalence of *Sarcocystis* was determined by peptic digestion and Giemsa staining. When analysing the diaphragm, heart, oesophagus, and tongue muscle samples of sheep from China, Hu et al. (2017) [4] identified the highest infection rates of *S. tenella* and *S. arieticanis* in the oesophagus, at 84.88% (73/86) and 51.16% (44/86), respectively. The examination of diaphragm, heart, oesophagus, and tongue muscle samples from 175 sheep from Egypt [14] showed the highest prevalence of *S. tenella* and *S. arieticanis* in the oesophagus (62.29% and 48.00%) and diaphragm (33.71% and 24.00%). Interestingly, in two reports, no microcysts of *S. arieticanis* were identified in the heart of domestic sheep [4,14]. The prevalence of *S. tenella* and *S. arieticanis* was established in these two studies by a microscopic analysis of 0.5 mm pieces of muscle squeezed between two glass slides. In summary, further molecular studies on the prevalence of *S. tenella* and *S. arieticanis* in various geographical regions are necessary to identify the distribution of these *Sarcocystis* species in different organs.

## 5. Conclusions

Based on digestion, nPCR, and subsequent sequencing, the presence of two *Sarcocystis* species, *S*. *arieticanis* and *S*. *tenella*, was confirmed in diaphragm, oesophagus, and heart muscle samples of sheep raised in Lithuania. This is the first identification of *Sarcocystis* species in sheep from Lithuania. Both detected species are transmitted by canids. Furthermore, twenty sarcocysts were excised from muscle tissues and characterised at *cox1*. The sequence comparison showed relatively high intraspecific genetic variation; thus, for the identification of *S*. *arieticanis* and *S*. *tenella*, it is important to accumulate *cox1* sequences of these two species originating from different geographical areas and hosts.

The detection rates of *S*. *arieticanis* and *S*. *tenella* in diaphragm, oesophagus, and heart samples varied from 82.61% to 100%. Significant differences were observed among the frequencies of detection of *S*. *tenella* in the three muscle types analysed (χ^2^ = 14.80, df = 2, *p* < 0.01), whereas such differences were not established in the case of *S*. *arieticanis* (χ^2^ = 1.28, df = 2, *p* = 0.575). Microscopical studies conducted in Saudi Arabia, China, and Egypt also reported considerable differences in the prevalence of *Sarcocystis* spp. in different organs [4,14,15]. The reasons for the uneven distribution of *Sarcocystis* species in different muscle types of sheep have yet to revealed.

## Figures and Tables

**Table 1:** Oligonucleotide primers used to amplify the *cox1* region of *Sarcocystis* spp. from domestic sheep.

*Sarcocystis* Species	Primer Name	Orientation	Primer Sequence	Ta, °C	Length of PCR Product, bp
*S. arieticanis*	SF1 ^1^	Forward	ATGGCGTACAACAATCATAAAGAA	53	913
	SsunR3 ^PS^	Reverse	CCGTTGGWATGGCRATCAT		
	V2arie3 ^PS^	Forward	TAGTTCTTGGCCTGGCTATTCTT	60	371
	V2arie4 ^PS^	Reverse	CTGACCTCCAAAAACTGGCTTAC		
*S. gigantea*	V2gig1 ^PS^	Forward	GCACTTCGAGCATTCTTGG	57	548
	V2gig2 ^2^	Reverse	ATCTACATCCACCGTAGGAACCTTA		
	V2gig3 ^2^	Forward	CAGCAAGTACCAAGTTCTGTACGTC	62	322
	V2gig4 ^PS^	Reverse	GGTGCCGAGTACCGAGATACAT		
*S. medusiformis*	V2medu1 ^PS^	Forward	TTAATGGCATATCGTACTACCTATTG	56	729
	V2medu2 ^PS^	Reverse	CCCATGCATCAACCTCCAG		
	V2medu3 ^PS^	Forward	GTATCCTGGGGGCCATTAACTT	61	389
	V2medu4 ^PS^	Reverse	CCAAACCAGTGTTCCGAGTATTG		
*S. mihoensis*	V2miho1 ^PS^	Forward	ATCTTTACACTGCACGGTTTGTTT	60	844
	V2miho2 ^PS^	Reverse	AGTCGTTATGTCGGAAGTCAACAG		
	V2miho3 ^PS^	Forward	GATGTTACCTCGGGTAAATGCTCTT	60	526
	V2miho4 ^PS^	Reverse	AAAAACATGTCTAGCTCCTAACACC		
*S. tenella*	SF1 ^1^	Forward	ATGGCGTACAACAATCATAAAGAA	53	913
	SsunR3 ^PS^	Reverse	CCGTTGGWATGGCRATCAT		
	V3tenF3 ^PS^	Forward	ACGCTATTTACCTGGGCAATC	59	381
	V3tenR2 ^PS^	Reverse	TAGTCACGGCAGAGAAGTAGGAC		

^1^ Ref. [24]. ^2^ Ref. [25]. ^PS^ Present study. Ta primer annealing temperature.

**Table 2:** Intraspecific genetic variation of *S*. *arieticanis* and *S*. *tenella*.

Sequence Similarity, %	Intermediate Host	Country	NCBI GenBank Acc. No.	Reference
*S*. *arieticanis*				
99.33–99.78	*Ovis aries*	Spain	MK419975–MK419976	[7]
98.77–99.11	*Ovis aries*	China	MF039324	[4]
92.39–93.85	*Ovis aries*	Egypt	MH413047–MH413048	[14]
*S*. *tenella*				
97.77–99.89	*Ovis aries musimon*	Austria	MW768881–MW768899	[25]
98.76–99.78	*Rupicapra rupicapra tatrica*	Poland	KP263744–KP263751	[28]
98.66–99.78	*Ammotragus lervia*	Spain	MW848314–MW848319	[29]
98.43–99.78	*Ovis aries*	Spain	MK419977–MK420010	[7]
98.21–99.78	*Ovis aries*	Norway	KC209723–KC209732	[24]
98.96–99.65	*Ovis ammon*	China	MH561854	[9]
98.55–99.55	*Ovis aries*	India	MH523439–MH523443	[3]
97.32–98.10	*Ovis aries*	China	MF039322–MF039323	[4]
95.86–97.54	*Ovis aries*	Egypt	MH413045–MH413046	[14]

**Table 3:** The identification of *S*. *arieticanis* and *S*. *tenella* using the nPCR method applied in the present study.

Species	Sequence Similarity, %		
	Intraspecific Variation		Interpsecific Variation
	Comparison between Isolates Obtained in the Present Study	Comparison of Sequences from the Same Species Available in Genbank	
*S. arieticanis*	97.54–100	91.36–99.69	86.83–88.09 *S*. *hircicanis*, 77.35–79.30 *S*. *cervicanis*, 76.47–78.57 *S*. *capracanis*
*S*. *tenella*	97.93–100	95.27–100	89.05–92.63 *S*. *capracanis*, 86.73–87.91 *S*. *heydorni*, 81.55–84.52 *S*. *gracilis*

**Table 4:** The distribution of *S*. *arieticanis* and *S*. *tenella* in different muscle types of sheep raised in Lithuania.

Positive Cases of *Sarcocystis* spp.	Muscle Type		
	Diaphragm	Oesophagus	Heart
*Sarcocystis* spp.	69/69 (100 %)	67/69 (97.10 %)	52/52 (100 %)
*S*. *arieticanis* overall	65/69 (94.20 %)	63/69 (91.30 %)	46/52 (88.46 %)
*S*. *tenella* overall **	69/69 (100 %) ^a,^***	57/69 (82.61 %) ^b,^***	49/52 (94.23 %)
*S*. *arieticanis* single infection	0/69 (0 %)	10/69 (14.49 %)	3/52 (5.77 %)
*S*. *tenella* single infection	4/69 (5.80 %)	4/69 (5.80 %)	6/52 (11.54 %)
Mixed infections with *S*. *arieticanis* and *S*. *tenella* *	65/69 (94.20 %) ^c,^**	53/69 (76.81 %) ^d,^**	43/52 (82.69 %)
The prevalence of *Sarcocystis* spp. depending on the age group of sheep			
*S*. *arieticanis* in sheep younger than two years	42/46 (91.3 %)	41/46 (89.1 %)	29/31 (93.5 %)
*S*. *arieticanis* in sheep older than two years	23/23 (100 %)	22/23 (95.7 %)	17/21 (81.0 %)
*S*. *tenella* in sheep younger than two years	46/46 (100 %)	39/46 (84.8 %)	30/31 (96.8 %)
*S*. *tenella* in sheep older than two years	23/23 (100 %)	18/23 (78.3 %)	19/21 (90.5 %)

^a^ > ^b^, ^c^ > ^d^. * *p* < 0.05, ** *p* < 0.01, *** *p* < 0.001.

## Data Availability

The *cox1* sequences of *S*. *arieticanis* and *S*. *tenella* were submitted to the GenBank database under the accession numbers ON858956–ON859017.
